# Supplementary material for: Uncovering the boundaries of Campylobacter species through large-scale phylogenetic and nucleotide identity analyses
Source: mSystems. 2024 Mar 26;9(4):e01218-23. doi: 10.1128/msystems.01218-23 (PMC11019964; doi:10.1128/msystems.01218-23)
Supplement: Figure S1 — Core genome phylogenetic tree comparison with 95% ANI clustering. [file msystems.01218-23-s0001.pdf]

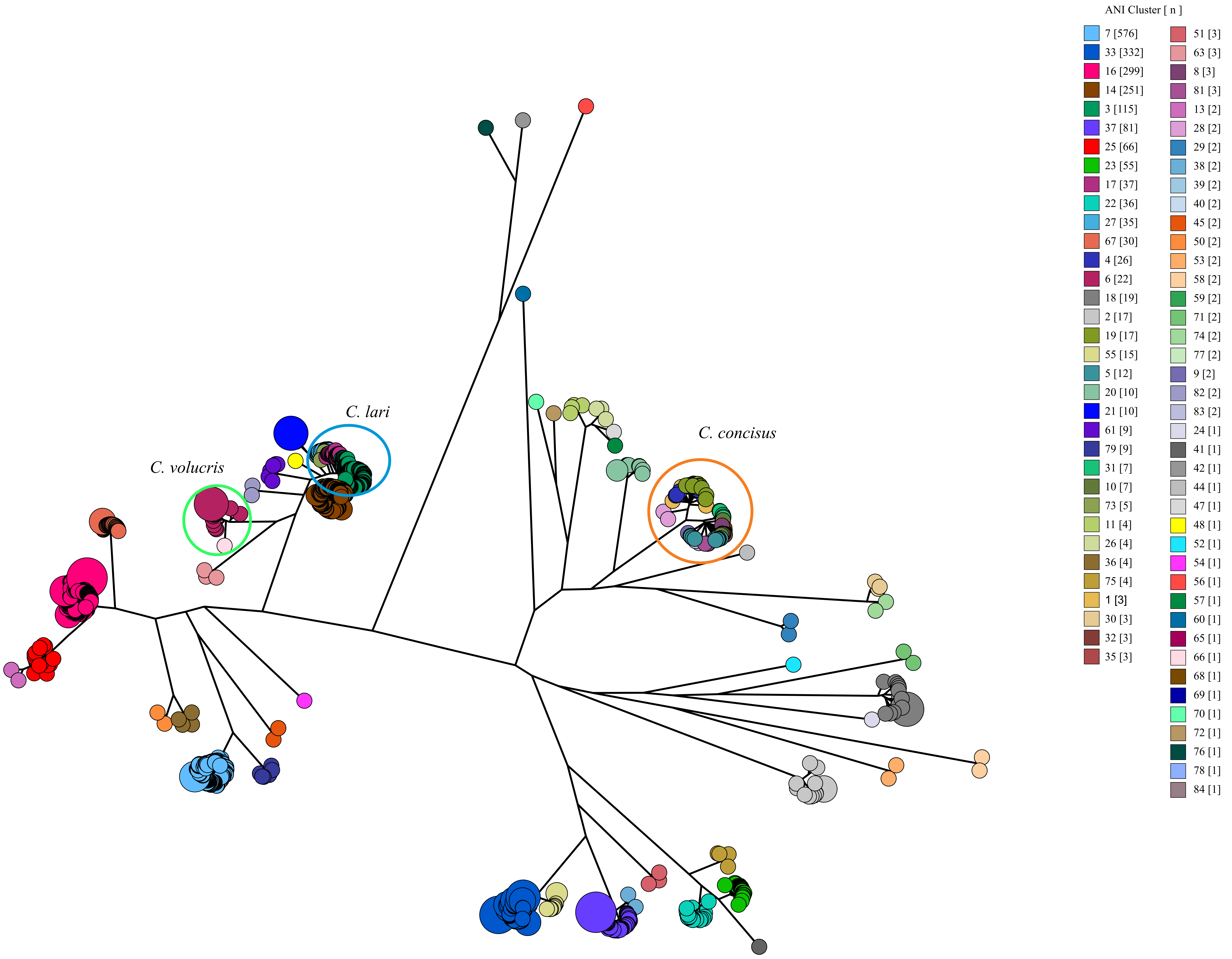

Supplementary Figure 1. **Core genome phylogenetic tree of 2193 genomes overlaid with 84 ANI clusters at 95% cut-off.** The tree was the same as Figure 1. The ANI clusters were obtained from average linkage clustering of ANI dissimilarity from 8440 Campylobacter genomes at 95% ANI. The *C. concisus*, *C.lari* and *C. volucris* branch were devided into numerous small ANI clusters at this cut-off.
