# Supplementary material for: Uncovering the boundaries of Campylobacter species through large-scale phylogenetic and nucleotide identity analyses
Source: mSystems. 2024 Mar 26;9(4):e01218-23. doi: 10.1128/msystems.01218-23 (PMC11019964; doi:10.1128/msystems.01218-23)
Supplement: Supplemental tables — Tables S1 and S2. [file msystems.01218-23-s0002.docx]

**Supplementary Table 1. Datasets used in this study.**

| Datasets | Number of complete genomes | Number of assembled genomes | Total |
| --- | --- | --- | --- |
| Core genome phylogeny dataset | 485 | 1708 | 2193 |
| ANI analysis dataset | 498 | 7942 | 8440 |

**Supplementary Table 2. 60 *Campylobacter* ANI genomic species and medoid genomes.**

| *Campylobacter* ANI genomic species | Medoid genome | NCBI user submitted denomination | ANI genomic species |
| --- | --- | --- | --- |
| 1 | SRR3217336 | *C. lari* | *Campylobacter novel genomic species 1* |
| 2 | SRR3658663 | *C. concisus* | *C. concisus* |
| 3 | SRR9962195 | *C. jejuni* | *C. jejuni* |
| 4 | SRR11267952 | *C. volucris* | *C. volucris* |
| 5 | SRR1995840 | *C. sputorum* | *C. sputorum* |
| 6 | CP049234.1 | *C. concisus* | *Campylobacter novel genomic species 2* |
| 7 | SRR11574529 | *C. upsaliensis* | *C. upsaliensis* |
| 8 | ERR460868 | *C. hyointestinalis* | *C. hyointestinalis* |
| 9 | CP012548.1 | *C. pinnipediorum* | *C. pinnipediorum* |
| 10 | SRR1995987 | *C. curvus* | *C. curvus* |
| 11 | CP085962.1 | *C. rectus* | *C. rectus* |
| 12 | ERR4045184 | *C. jejuni* | *Campylobacter novel genomic species 3* |
| 13 | SRR17258904 | *C. coli* | *Campylobacter novel genomic species 4* |
| 14 | SRR5808786 | *C. lari* | *C. lari* |
| 15 | ERR4224434 | *C. coli* | *Campylobacter novel genomic species 5* |
| 16 | ERR987477 | *C. fetus* | *C. fetus* |
| 17 | CP053842.1 | *C. corcagiensis* | *C. corcagiensis* |
| 18 | ERR976084 | *Unspecified Campy* | *Campylobacter novel genomic species 6* |
| 19 | SRR891413 | *C. ureolyticus* | *C. ureolyticus* |
| 20 | SRR9962571 | *C. jejuni* | *Campylobacter novel genomic species 7* |
| 21 | SRR3216181 | *C. lari* | *C. subantarcticus* |
| 22 | ERR670757 | *C. lanienae* | *C. lanienae* |
| 23 | ERR670749 | *C. lanienae* | *Campylobacter novel genomic species 8* |
| 24 | CP053841.1 | *C. blaseri* | *C. blaseri* |
| 25 | SRR8272101 | *C. coli* | *C. coli* |
| 26 | ERR1725009 | *C. showae* | *Campylobacter novel genomic species 9* |
| 27 | SRR1995159 | *C. lari* | *Campylobacter novel genomic species 10* |
| 28 | SRR3658649 | *C. concisus* | *Campylobacter novel genomic species 11* |
| 29 | CP012542.1 | *C. mucosalis* | *C. mucosalis* |
| 30 | SRR10390898 | *C. armoricus* | *C. armoricus* |
| 31 | CP007770.1 | *C. insulaenigrae* | *C. insulaenigrae* |
| 32 | CP049075.1 | *Candidatus Campylobacter infans* | *Campylobacter novel genomic species 12* |
| 33 | SRR8101494 | *C. hepaticus* | *C. hepaticus* |
| 34 | SRR8731332 | *C. jejuni* | *Campylobacter novel genomic species 13* |
| 35 | CP012552.1 | *Campylobacter sp. RM16192* | *Campylobacter novel genomic species 14* |
| 36 | CP053845.1 | *Campylobacter sp. CCUG 57310* | *Campylobacter novel genomic species 15* |
| 37 | ERR473921 | *Campylobacter sp.RM6137* | *Campylobacter novel genomic species 16* |
| 38 | CP059599.1 | *Campylobacter sp. RM5004* | *Campylobacter novel genomic species 17* |
| 39 | SRR10248245 | *C. helveticus* | *C. helveticus* |
| 40 | CP022347.1 | *C. avium* | *C. avium* |
| 41 | CP007766.1 | *C. peloridis* | *C. peloridis* |
| 42 | CP059600.1 | *Campylobacter sp. RM12651* | *Campylobacter novel genomic species 18* |
| 43 | ERR1512603 | *C. coli* | *Campylobacter novel genomic species 19* |
| 44 | CP012545.1 | *Campylobacter sp. RM6914* | *Campylobacter novel genomic species 20* |
| 45 | CP063091.1 | *C. cuniculorum* | *C. cuniculorum* |
| 46 | SRR4067225 | *C. jejuni* | *Campylobacter novel genomic species 21* |
| 47 | CP012544.1 | *C. showae* | *C. showae* |
| 48 | CP053848.1 | *C. ornithocola* | *C. ornithocola* |
| 49 | CP007769.1 | *Campylobacter sp. RM16704* | *Campylobacter novel genomic species 22* |
| 50 | ERR976078 | *Unspecified Campylobacter* | *Campylobacter novel genomic species 23* |
| 51 | CP010995.1 | *C. iguaniorum* | *C. iguaniorum* |
| 52 | CP053844.1 | *C. geochelonis* | *C. geochelonis* |
| 53 | CP000776.1 | *C. hominis* | *C. hominis* |
| 54 | CP076657.1 | *C. novaezeelandiae* | *C. novaezeelandiae* |
| 55 | SRR3214524 | *C. fetus* | *Campylobacter novel genomic species 24* |
| 56 | CP035946.1 | *C. canadensis* | *C. canadensis* |
| 57 | SRR616246 | *C. showae* | *Campylobacter novel genomic species 25* |
| 58 | CP085963.1 | *C. gracilis* | *C. gracilis* |
| 59 | CP063089.1 | *Campylobacter sp. 2014D-0216* | *Campylobacter novel genomic species 26* |
| 60 | AP024713.1 | *Campylobacter sp. 19-13652* | *Campylobacter novel genomic species 27* |

The last column shows the output of CampyGStyper python script.
